# Comprehensive characterization of *PKHD1* mutation in human colon cancer

**DOI:** 10.1002/cam4.6796

**Published:** 2024-01-04

**Authors:** Lu Han, Fangming Gong, Xuxiaochen Wu, Wanxiangfu Tang, Hua Bao, Yue Wang, Daizhenru Wang, Yulan Sun, Peng Li

**Affiliations:** ^1^ Department of Oncology The First Medical Center, PLA General Hospital Beijing China; ^2^ Department of General Surgery The First Medical Center, PLA General Hospital Beijing China; ^3^ Geneseeq Research Institute, Nanjing Geneseeq Technology Inc. Nanjing China; ^4^ Shandong Cancer Hospital and Institute, Shandong First Medical University and Shandong Academy of Medical Sciences Jinan China

**Keywords:** biomarkers, colon cancer, mutations, tumor‐infiltration immune cell

## Abstract

**Introduction:**

The *PKHD1* (Polycystic Kidney and Hepatic Disease 1) gene is essential for producing fibrocystin or polyductin, which is crucial in various cellular functions. Mutations in *PKHD1* have been found to be involved in the development and progression of colorectal cancer (CRC). Along with *APC*, *TP53*, and *KRAS*, *PKHD1* is one of the most frequently mutated genes in CRC. *PKHD1* expression is governed by the Wnt/PCP pathway, often dysregulated in CRC. Targeting this pathway, crucial for CRC progression, could unveil potential therapeutic strategies for colon cancer treatment.

**Methods:**

This study examined an in‐house dataset of 3702 colon cancer samples, analyzing mutation landscapes, clinical features, tumor mutational burden (TMB), microsatellite instability (MSI), and chromosomal instability (CIN) score. For the survival analysis of *PKHD1* patients, survival data of 436 colon adenocarcinoma samples were obtained from TCGA dataset. Additionally, 433 samples from TCGA with RNA‐seq data were used for the assessment of immune cell infiltration and gene set enrichment analysis.

**Results:**

Polycystic Kidney and Hepatic Disease 1 mutation was detected in 424 colon cancer patients from our in‐house cohort and was associated with increased TMB, higher MSI, and lower CIN score. Importantly, within the TCGA dataset, *PKHD1* mutations were identified as an independent prognostic factor, not merely correlated with established prognostic biomarkers, and were associated with poorer overall survival outcomes. In terms of immune response, these mutations correlated with increased enrichment scores for 12 immune cell types, including B cell plasma, macrophages, and naive CD4+ T cells. Additionally, interferon alpha and interferon‐gamma gene sets were significantly down‐regulated in patients with *PKHD1* mutations (FDA *q*‐value < 0.1).

**Conclusions:**

Overall, these findings suggest that *PKHD1* may be a potential biomarker for the prognosis of colon cancer and provide some insight for personalized immunotherapy.

## Graphical abstract

In this study of 3702 samples, mutations in the PKHD1 gene, frequently observed in colorectal cancer (CRC), were correlated with increased tumor mutational burden and microsatellite instability, but decreased chromosomal instability. These mutations also serve as an independent prognostic biomarker, associated with poorer overall survival outcomes in TCGA COAD patients. These critical findings reinforce the potential of PKHD1 as a pivotal biomarker for colon cancer prognosis and may facilitate the emergence of personalized immunotherapy strategies.

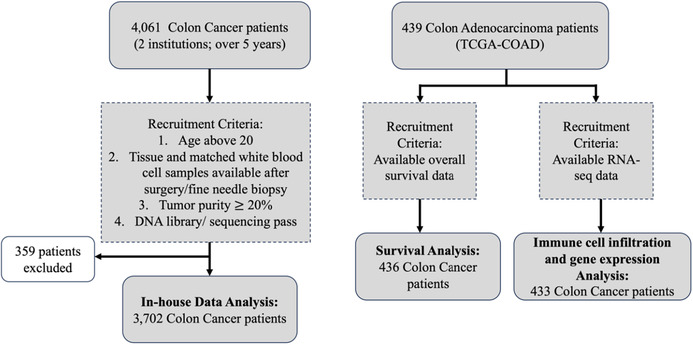

## 1 INTRODUCTION

Colorectal cancer (CRC) ranks as the third highest cause of cancer‐related deaths worldwide, with an estimated 52,550 deaths in 2023.^1^ Despite significant advancements in CRC screening and prevention over the past decade, diagnosis at an early stage—specifically stages 1 and 2—occurs in only 30%–40% of patients. These early‐stage patients have an estimated five‐year survival rate of 90%. On the other end of the spectrum, 20% of patients are diagnosed at stage 4, where the cancer has metastasized; the five‐year survival rate for this group drastically drops to 12.5%.^2^, ^3^ Even though the mortality rate of CRC has consistently declined by roughly 20% in the last decade, there has been a rise in the occurrence rate in younger individuals.^4^ The incidence rate among younger adults increased by approximately 2.2% per year between 2010 and 2017.^5^ The rising incidence trend among younger people highlights the importance of continued monitoring of trends, promotion of screening and prevention strategies, and improvement of therapeutic options to reduce the burden of CRC.

For a long time, surgery and chemotherapy have been used as the major therapeutic strategies for the treatment of CRC. However, due to the ineffective detection of CRC in the early stage, the prognosis of this strategy for patients with metastatic lesions could be more satisfactory. As a new treatment, targeted therapy controls tumor growth by blocking key pathways and immune checkpoints with targeted drugs such as bevacizumab.^6^ Hence, identifying and characterizing novel CRC‐associated genes could help optimize the clinical diagnosis and offer insights into new, targeted therapeutic approaches to CRC.

The *PKHD1* gene (Polycystic Kidney and Hepatic Disease 1) is responsible for producing the fibrocystin protein that plays an essential role in multiple cellular functions, such as cell adhesion, signal transduction, and regulation of the cell cycle. Along with *APC*, *TP53*, and *KRAS*, *PKHD1* is one of the most oftentimes mutated genes in CRC.^7^ *PKHD1* expression is regulated by the non‐canonical Wnt/Planar Cell Polarity (PCP) signaling pathway, which is shown to be dysregulated in CRC.^8^, ^9^ Blocking the Wnt signaling pathway, which is crucial for the development of colon cancer, is a therapeutic strategy for treating CRC.^10^ Furthermore, the decreased expression of the *PKHD1* gene could serve as a potential biomarker for the diagnosis and prognosis of CRC. Specifically, patients presenting with lower levels of *PKHD1* expression may potentially demonstrate a higher likelihood of CRC development or a more severe prognosis.^2^ A comprehensive investigation of CRC patients harboring *PKHD1* mutations is currently lacking in terms of detailed clinical and pathological profiles. Therefore, further research is warranted, focusing on CRC patients carrying *PKHD1* mutations to gain a more holistic understanding of *PKHD1*'s role in CRC pathogenesis and to fill the gap in the current knowledge.

While colorectal malignancies encompass both colon and rectal cancers, each with distinct histopathology and molecular profile.^11^ Given the data available, our study primarily focuses on colon cancer. We analyzed two colon cancer cohorts to discover the genetic and clinicopathological features of *PKHD1*‐mutated patients—data obtained from our in‐house clinical sequencing database and the Cancer Genome Atlas (TCGA). We captured data on colon cancer patients with and without *PKHD1* alterations identified in tumors, which allows us to assign specific patterns of mutations as a consequence of sex, age, microsatellite instability (MSI), chromosomal instability (CIN), tumor mutational burden (TMB), survival, immune cell infiltration, and gene set enrichment with other driver mutations.

## 2 METHOD

### 2.1 Sample collection

Samples from 3702 colon cancer patients, including tumor FFPE and paired normal whole blood samples, were collected over 5 years from The First Medical Center, PLA General Hospital, Beijing, China and Shandong Cancer Hospital and Institute, Shandong First Medical University and Shandong Academy of Medical Sciences, Jinan, China. The sequencing and profiling of the samples were conducted at Geneseeq Technology Inc., a laboratory certified by the Clinical Laboratory Improvement Amendments (CLIA) and accredited by the College of American Pathologists (CAP). The ethics boards approved the study of the respective institutions and are in compliance with the Declaration of Helsinki. Prior to sample collection, all patients were fully informed and consented to participate in the research.

### 2.2 DNA library preparation

As per the previous literature,^12^ the DNA extraction and sequencing libraries were prepared. Genomic DNA was extracted from the fresh tumor or formalin‐fixed paraffin‐embedded tissue samples, and normal control samples were collected from peripheral whole blood. Customized xGen lockdown probes were designed as per the guidelines provided by Integrated DNA Technologies. Subsequently, the libraries were quantified using qPCR with the KAPA Library Quantification Kit by KAPA Biosystems, and the fragment size was determined with the Agilent 2100 Bioanalyzer.

### 2.3 Sequencing and somatic mutation calling

The GeneseeqPrime panel (Nanjing Geneseeq Technologies Inc.) was used to target the sequencing of 437 cancer‐related genes. Samples were sequenced on Illumina HiSeq4000, followed by removing low‐quality regions in sequenced reads using Trimmomatic (v0.39). The reads were then aligned to the hg19 human reference genome using Burrows–Wheeler Aligner (v0.7.12). Further processing of the trimmed and aligned reads, including deduplication, local realignment around InDels, and base quality score recalibration, was carried out using tools from best practices in Genome Analysis Toolkit v3.4.0. The VarScan2 tool was used to call for somatic mutations. To ensure accuracy, only results with at least 2% variant allele frequencies and five or more supporting reads were kept for further analysis. Furthermore, an internally generated list of known sequencing artifacts was referenced against all mutations. Any matches were subsequently removed based on normal control samples.

The assessment of TMB was conducted based on counting the number of somatic, coding, base substitution, and indel mutations (excluding known driver mutations) per megabase of genome examined as previously described.^13^ The microsatellite (MS) status was examined through a specialized analysis algorithm.^13^ Briefly, 52 mononucleotide repeats with a minimum of 15 bp repeats were identified as MSI sites within the GeneseeqPrime panel. Sequencing reads for these sites were counted and compared to normal samples. A site was labeled as unstable if its length distribution significantly differed. A sample was categorized as MSI if more than 40% of these evaluated sites showed instability. The CIN was determined as the average proportion of all segments with a log2 ratio either exceeding 0.2 or falling below −0.2. Assay validations of mutation calling, MSI, TMB, and CIN determination were performed with CLIA/CAP accreditation.

### 2.4 Survival analysis

To assess the prognostic potential of *PKHD1* mutations in colon cancer patients, we enriched our analysis by incorporating the survival data of a cohort consisting of 436 colon adenocarcinoma (COAD) patients with tumor tissue samples and matched blood‐derived normal samples, which was retrieved from cBioPortal (https://www.cbioportal.org/study/summary?id=coadread_tcga_pan_can_atlas_2018).

### 2.5 Immune cell infiltration and gene expression analysis

The RNA‐Seq data of the TCGA COAD cohort (*N* = 433) was acquired. Samples were classified based on the presence or absence of *PKHD1* mutations. Using xCELL,^14^ immune cell infiltration was estimated. Gene set enrichment analysis (GSEA) was performed on a set of 50 HALLMARK genes from the Human MSigDB collection using the GSEA software (v4.3.0). The analysis was run with 1000 permutations and CHIP annotation using “Human_Gene_Symbol_with_Remapping_MSigDB.v2022.1.Hs.chip”. The permutation type was set to “phenotype,” and all other parameters were set to default.

### 2.6 Statistical analysis

The statistical analysis in this study was performed using R (v4.2.2). The following tests were used: Mann–Whitney *U* test for age, TMB, CIN, MSI score, and xCell score; Fisher's exact test for sex proportions; chi‐square for association between cancer stage and *PKHD1* mutation; and Cox proportional hazards regression model for univariate and multivariate analysis of prognostic factors. A *p*‐value of 0.05 or less was considered statistically significant. The Kaplan–Meier survival plots were generated using the “survival” and “survminer” packages in R.

## 3 RESULTS

### 3.1 Clinicopathological characteristics

**FIGURE 1:**
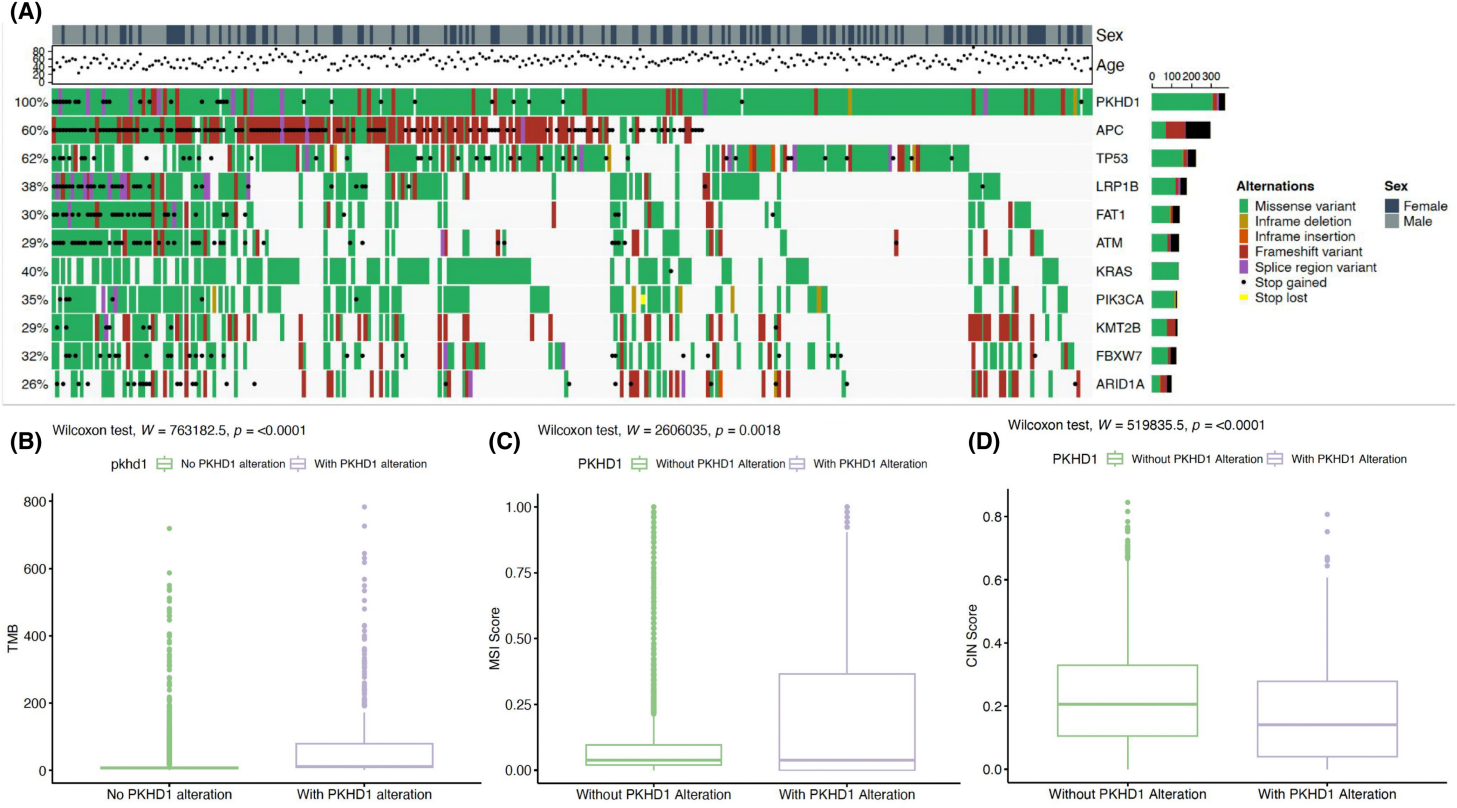
Genomic landscape and biomarkers of colon cancer patients with *PKHD1* mutations. (A) The oncoprint of co‐occurring somatic mutations in patients with *PKHD1* mutations. Genes are ordered by prevalence in the cohort. (B) TMB comparison between *PKHD1* and wild‐type groups. (C) MSI comparison between *PKHD1* and wild‐type groups. (D) CIN comparison between *PKHD1* and wild‐type groups. All TMB are calculated using non‐synonymous mutations and undergoing log2 transformation. Significance was determined using Wilcoxon signed‐rank test.

We analyzed the sequencing data for 3702 colon cancer samples (Figure S1). The most mutated genes in this cohort included *TP53*, *APC*, *KRAS*, *SMAD4*, and *PIK3CA*, accounting for 73%, 57%, 40%, 19%, and 20% of total nonsynonymous mutations, respectively (Figure S2A). The gender distribution of colon cancer patients was also analyzed, with male patients accounting for 59% of the cohort (Table S1). The study found a 2.4% increase in male patients with *PKHD1* alterations (Figure S3). The average age distribution of colon cancer patients was around 60, and the average age of patients with *PKHD1* alterations was slightly lower, but there was no significant difference (*p* = 0.16) (Figure S2B). A notable observation was the prevalence of *PKHD1* mutations in the early stages of colon cancer (Figure S2C; Table S3), a trend that was corroborated by data from the TCGA COAD cohort (Table S4). Additionally, we identified 424 patients with *PKHD1* mutations in the cohort and analyzed their genomic landscape (Figure 1A). These patients showed higher TMB (*p* < 0.0001, Figure 1B), higher MSI score (*p* = 0.0018, Figure 1C), and lower CIN score (*p* < 0.0001, Figure 1D). Regarding the co‐occurrence of the analyzed genes, *TP53* showed a significant mutually exclusive relationship with other genes, meaning that mutations in *TP53* were less likely to occur together with mutations in other genes. The co‐occurrence value of *APC*, *KRAS*, and *SMAD4* was significantly lower than other genes, indicating moderate mutual exclusivity (Figure S4).

### 3.2 *PKHD1* mutated patients showed worse overall survival

**FIGURE 2:**
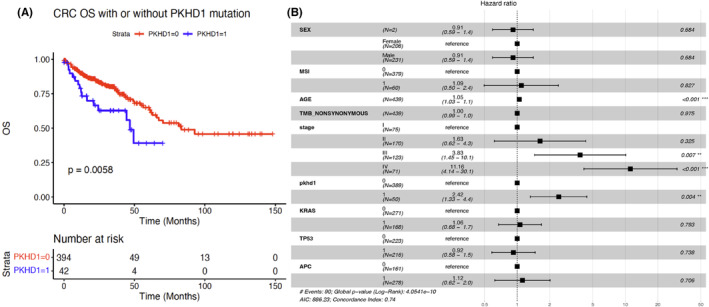
Comparison of overall survival between *PKHD1* and wild‐type groups. (A) Kaplan–Meier survival curve for overall survival of *PKHD1*‐mutated versus wild‐type patients. The 0 denoted *PKHD1* wild type, and 1denoted PKHD1 mutation. (B) Multivariate Cox analysis of *PKHD1* mutation presence in addition to sex, age, stage, MSI, TMB, and prognostic biomarkers.

The current study investigated a cohort of 436 colon cancer patients and revealed that individuals with *PKHD1* somatic mutations experienced significantly poorer overall survival outcomes compared to those without the mutation (*p* = 0.0058, Figure 2A). In addition, this study conducted both univariate and multivariate Cox analysis to determine the significance of the *PKHD1* gene mutation status in relation to other factors affecting overall survival, such as sex, MSI, age, TMB, and stage of cancer. The results showed that the *PKHD1* gene mutation status was significant in the univariate (Table S2) and multivariate Cox analysis (Figure 2B). Additionally, age and stage were found to be the significant factors affecting overall survival (*p* < 0.001, Figure 2B). Further analysis was conducted to investigate the co‐occurrence of *PKHD1* mutation with other gene mutations, such as *TP53*, *APC*, and *KRAS*. The study found that colon cancer patients with *PKHD1* mutation co‐occurring with *TP53*, *APC*, and *KRAS* had a worsening trend in overall survival outcomes (Figure S5A–C). Additionally, in the *PKHD1* WT group, TP53 mutation caused a dramatic decrease in the overall survival rate (*p* = 0.013, Figure S5A). However, in the *PKHD1* mutated group, both WT and mutated *KRAS* patients had significantly worse overall survival outcomes than the *PKHD1* WT patients (*p* = 0.015, Figure S5C). *PKHD1* somatic mutations may be a significant factor in determining overall survival outcomes in colon cancer patients, especially when co‐occurring with other gene mutations. However, age and stage remain the most significant factors affecting overall survival in colon cancer patients.

### 3.3 Immune cell infiltration and gene set enrichment analysis

**FIGURE 3:**
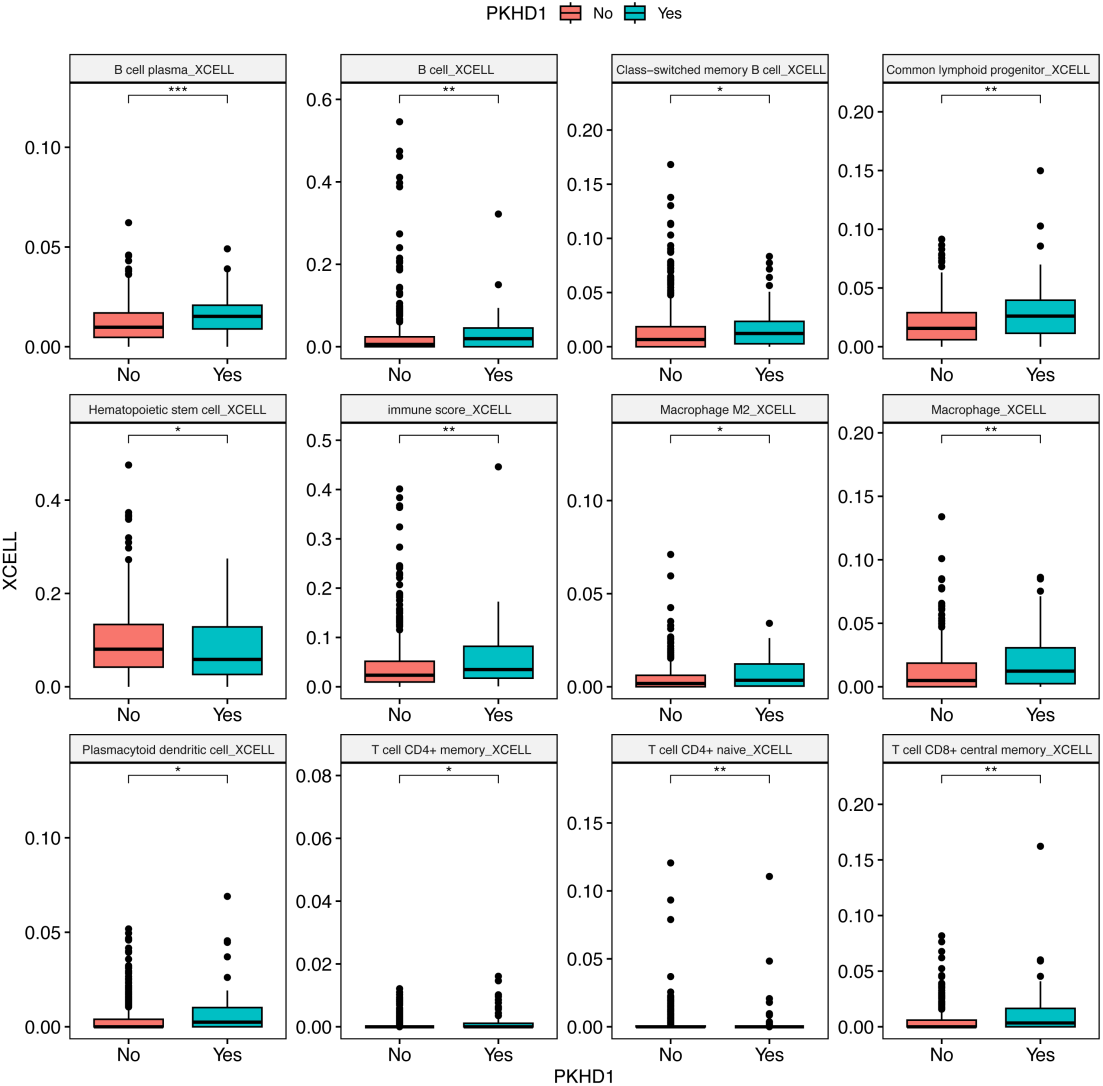
Comparison of immune and stromal cell infiltrating between *PKHD1* and wild‐type groups. Twelve cell types with significantly different xCell scores are shown. Significance was determined by Wilcoxon signed‐rank test. *** = <0.001; ** = <0.01; * = <0.05.

**FIGURE 4:**
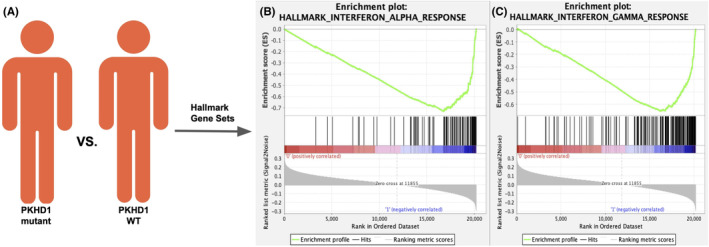
Comparison of gene set enrichment between *PKHD1* and wild‐type groups. (A) Schematic diagram comparing gene set enrichment between *PKHD1* mutant and wild‐type groups. Two gene sets with significantly different (FDA *q*‐value < 0.1) enrichment scores are shown: (B) Interferon Alpha and (C) Interferon Gamma are significantly down‐regulated in *PKHD1* samples.

This cohort study based on TCGA COAD cohort analyzed 433 patients with RNA sequencing data to investigate immune and stromal cell infiltration and gene set enrichment analysis in relation to the *PKHD1* mutation status. The cohort was divided into two groups: *PKHD1‐*mutated patients and wild‐type patients. We analyzed twelve different immune cell types and found that among them, *PKHD1* mutated patients had higher xCell scores for seven cell types: B cell plasma, B cell, common lymphoid progenitor, immune score, macrophage, T cell CD4+ naive cell, and T cell CD8+ central memory (Figure 3, Table S5). These findings suggest that these immune cell types have a stronger association with the overall immune and microenvironment scores compared to the other cell types. Furthermore, the study conducted GSEA and found that three hallmark gene sets were enriched in *PKHD1* mutated phenotype patients: interferon‐gamma response, interferon‐alpha response, and allograft rejection (Figure 4A,B, Table S5). This suggests that the *PKHD1* mutation may have an impact on the immune response and immune‐related pathways in colon cancer patients.

## 4 DISCUSSION

The results of this study indicate that *PKHD1* somatic mutations are associated with worse overall survival outcomes in colon cancer patients, particularly when co‐occurring with other gene mutations. Age and stage remain the significant factors affecting overall survival. These findings suggest *PKHD1* somatic mutations may be a potential prognostic biomarker in colon cancer patients.

Utilizing two separate cohort studies, the present study provides an initial analysis focusing on characteristics including sex, age, and stage of cancer in relation to *PKHD1* mutations in patients with colon cancer. We noted a higher frequency of *PKHD1* mutations during the early stages of colon cancer, with a particular prevalence at stage 2. Furthermore, molecular‐level analyses indicate that *PKHD1* mutations function as an independent prognostic biomarker for overall survival in colon cancer, distinct from well‐established factors such as TMB and MSI.^2^, ^15^ While age and stage emerged as the critical factors impacting overall survival, *PKHD1* somatic mutations correlated with worse overall survival outcomes both as independent mutations and when co‐existing with other gene mutations. Additionally, *PKHD1*‐mutated patients exhibited a unique immune and microenvironment profile, distinguished by increased xCell scores for specific immune cell types, such as B cell plasma, B cell, common lymphoid progenitor, and the enrichment of particular gene sets related to immune response.^14^ This study underscores the significance of genetic testing for colon cancer patients to detect mutations that could influence treatment choices and enhance outcomes. Moreover, these findings may hold implications for developing tailored immunotherapies for colon cancer patients based on their *PKHD1* mutation status.^16^

The most frequently mutated genes in colon cancer patients in this cohort were *TP53*, *APC*, *KRAS*, *SMAD4*, and *PIK3CA*, consistent with previous studies (Figure 1A). In addition, the study found that male patients had a higher incidence of *PKHD1* mutations, but there was no significant difference in the age distribution (Figure S1B). The co‐occurrence analysis of mutated genes showed that *TP53* had a significant mutually exclusive relationship with other genes, while *APC*, *KRAS*, and *SMAD4* had moderate mutual exclusivity (Figure S2). These findings provide a better understanding of the clinicopathological characteristics of *PKHD1* mutations in colon cancer patients and their co‐occurrence with other gene mutations.

We discovered that *PKHD1* somatic mutations were linked to poorer overall survival results, particularly when they appeared alongside other gene mutations like *TP53*, *APC*, and *KRAS* (Figure 2 and Figure S3A–C).^17^ Nonetheless, the multivariate Cox analysis identified age and stage as the most significant factors influencing overall survival (Figure 2B). Age and stage emerged as the primary determinants of overall survival outcomes, emphasizing the necessity of considering multiple factors when evaluating prognosis. The results suggest that while the *PKHD1* mutation status may serve as an important prognostic factor in colon cancer patients, it might not be an independent factor affecting overall survival outcomes.^18^, ^19^

Additionally, the study explored the immune and microenvironment profiles of *PKHD1*‐mutated patients through immune cell infiltration analysis. It was found that these patients had higher xCell scores for specific immune cell types and enrichment of particular gene sets related to immune response, such as B cell plasma, B cell, common lymphoid progenitor, immune score, macrophage, T cell CD4+ naive cell, and T cell CD8+ central memory (Figure 3, Table S5).^14^, ^20^ These findings may hold implications for developing personalized immunotherapies for colon cancer patients based on their *PKHD1* mutation status.^21^, ^22^ We hypothesize that *PKHD1‐*mutated patients with increased immune cell infiltration could benefit more from immunotherapy treatments.^23^

In conclusion, the findings suggest that *PKHD1* mutations act as an independent prognostic factor and are not merely correlated with the existence of already known prognostic factors, such as higher TMB and MSI scores and lower CIN scores. The study also highlights the prognostic significance of *PKHD1* mutations in colon cancer patients, especially when co‐occurring with other gene mutations. Finally, the study reveals the distinct immune and microenvironment profile of *PKHD1‐*mutated patients, which may have implications for developing personalized immunotherapies for colon cancer patients. Understanding the specific immune cell infiltration patterns and gene set enrichments in these patients can help identify potential therapeutic targets and enhance the efficacy of immunotherapy treatments. Future research should focus on elucidating the molecular mechanisms underlying the association between *PKHD1* mutations and immune response. This knowledge may facilitate the development of novel therapeutic strategies that maximize benefits for colon cancer patients with specific mutation profiles.

The current study could not thoroughly examine the impact of *PKHD1* on colon cancer due to incomplete clinicopathological information in the data sets used. The clinicopathological information was limited to basic parameters such as age, sex, and stage, excluding other potentially significant clinical features like location of tumor and grade. The study also faced challenges in thoroughly evaluating the repercussions of *PKHD1* mutations across the entire scope of CRC, primarily due to dataset constraints. Therefore, future studies with a more complete set of clinical and pathomorphological data spanning colon and rectal cancers are essential to more fully elucidating the significance of *PKHD1* mutations within the CRC landscape.

## AUTHOR CONTRIBUTIONS

**Lu Han:** Data curation (equal); formal analysis (equal); methodology (equal); validation (equal); writing – original draft (equal). **Fangming Gong:** Data curation (equal); formal analysis (equal); methodology (equal); validation (equal); writing – original draft (equal). **Xuxiaochen Wu:** Data curation (equal); formal analysis (equal); writing – review and editing (equal). **Wanxiangfu Tang:** Data curation (equal); formal analysis (equal); writing – review and editing (equal). **Hua Bao:** Data curation (equal); formal analysis (equal); writing – review and editing (equal). **Yue Wang:** Data curation (equal); formal analysis (equal); writing – review and editing (equal). **Daizhenru Wang:** Data curation (equal); formal analysis (equal); writing – review and editing (equal). **Yulan Sun:** Conceptualization (equal); supervision (equal). **Peng Li:** Conceptualization (equal); supervision (equal).

## CONFLICT OF INTEREST STATEMENT

Xuxiaochen Wu, Wanxiangfu Tang, Hua Bao, Yue Wang and Daizhenru Wang are employees of Nanjing Geneseeq Technology Inc., Nanjing, Jiangsu, China. The remaining authors have nothing to declare.

## ETHICS STATEMENT

All study protocols were approved by the Medical Ethics Committee of Nanjing Geneseeq Medical Laboratory (NSJB‐MEC‐2023‐07), and in accordance with international standards of good clinical practice. Written informed consents were provided by all patients.

## CONSENT FOR PUBLICATION

The content of this manuscript has not been previously published and is not under consideration for publication elsewhere.

## Supporting information


Appendix S1.

## Data Availability

The datasets used and/or analyzed during the current study are available from the corresponding author on reasonable request.
