# Supplementary material for: Comprehensive characterization of PKHD1 mutation in human colon cancer
Source: Cancer Med. 2024 Jan 4;13(1):e6796. doi: 10.1002/cam4.6796 (PMC10807659; doi:10.1002/cam4.6796)
Supplement: Supplementary file 1 — Appendix S1. [file CAM4-13-e6796-s001.docx]

Supplementary Material

Comprehensive characterization of *PKHD1* mutation in human colon cancer

**Lu Han^1*^, Fangming Gong^2*^, Xuxiaochen Wu^3^, Wanxiangfu Tang^3^, Hua Bao^3^, Yue Wang^3^, Daizhenru Wang^3^, Yulan Sun^4†^, Peng Li^2†^**

^1^Department of Oncology, The First Medical Center, PLA General Hospital, Beijing, China

^2^ Department of General Surgery, The First Medical Center, PLA General Hospital, Beijing, China

^3^ Geneseeq Research Institute, Nanjing Geneseeq Technology Inc., Nanjing, China

^4^ Shandong Cancer Hospital and Institute, Shandong First Medical University and Shandong Academy of Medical Sciences, Jinan, China

*: These authors contributed equally to this work and share first authorship

†: These corresponding authors contributed equally to this work

† **Correspondence:**

Peng Li, Ph.D.,

PLA General Hospital,

No.28 Fuxing 20 Road, Beijing 100853, China

Telephone: 086- 13522663625

E-mail: doctorlipeng@126.com

Yulan Sun, Ph.D.,

Shandong First Medical University and Shandong Academy of Medical Sciences,

NO.440 Jiyan Road, Jinan, Shandong 250117, China

Telephone: 086- 13583171910

E-mail: 306920775@qq.com

# Supplementary Figures and Tables

## Supplementary Figures


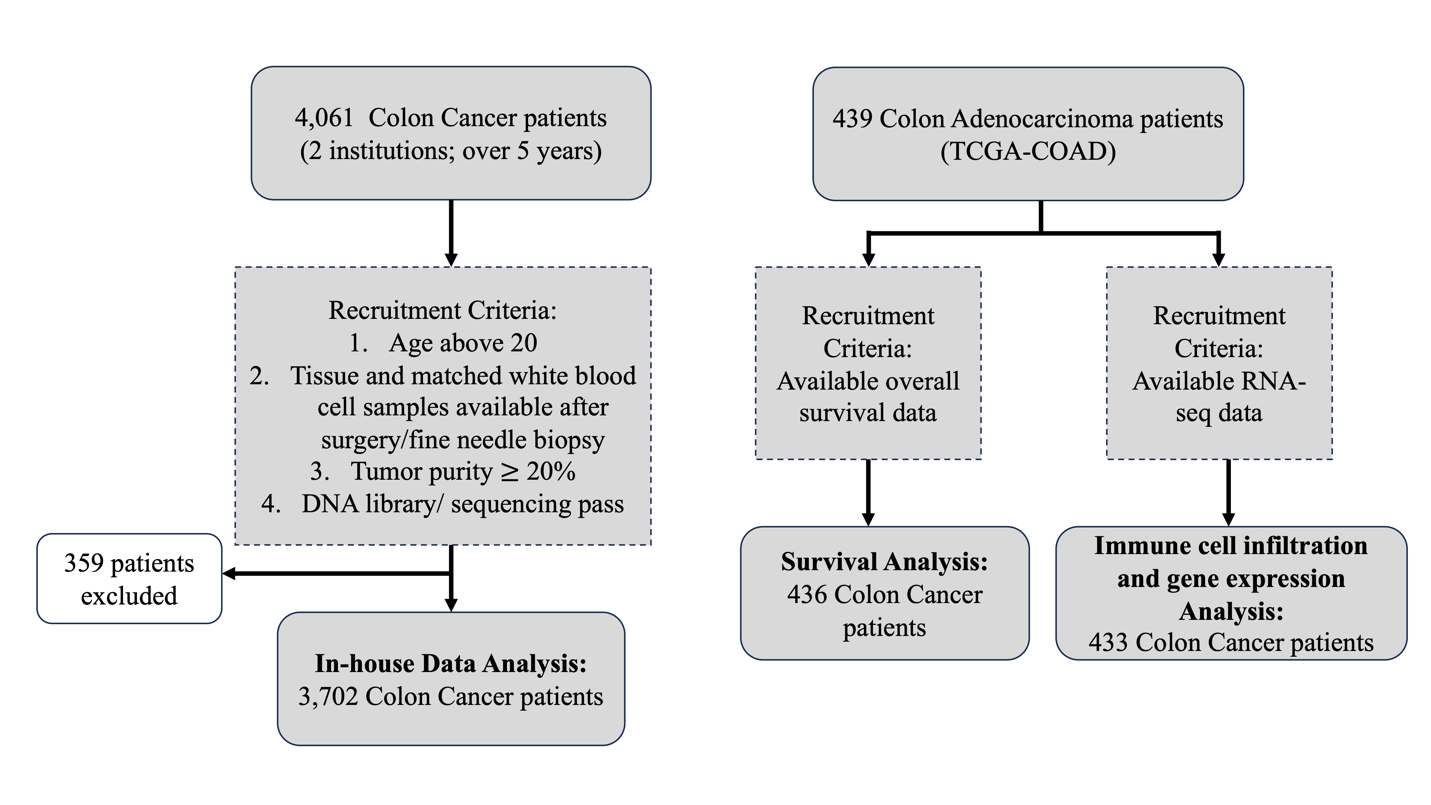


**Supplementary Figure 1. Schematic illustration depicting the study design.**


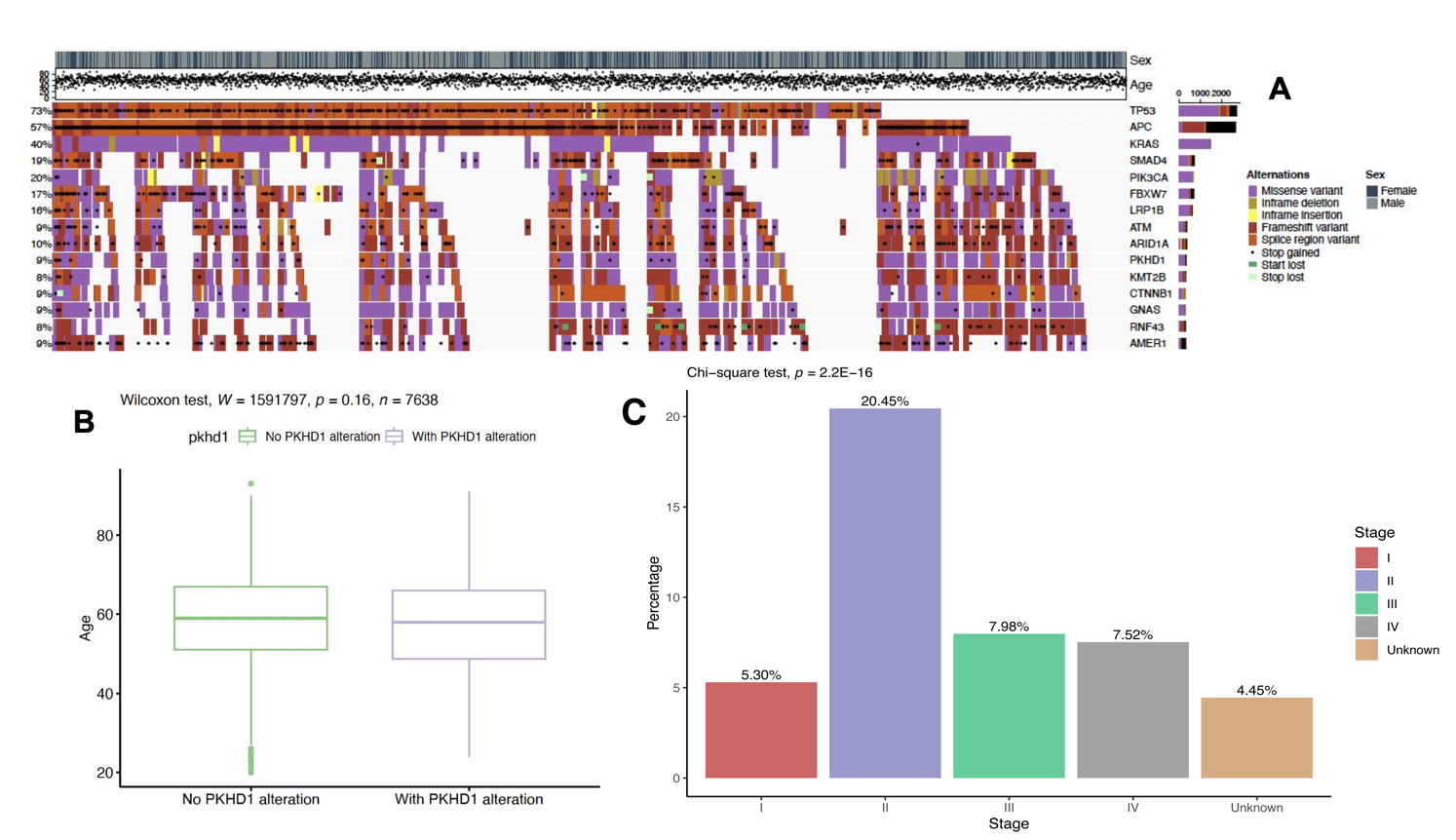


**Supplemental Figure 2.** Additional genomic and clinical landscape of colon cancer patients. A) Oncoprint of somatic mutations in top 15 genes in colon cancer patients. B) Age comparison between *PKHD1* and wild-type groups. C) Proportion of *PKHD1* mutant at each cancer stage.


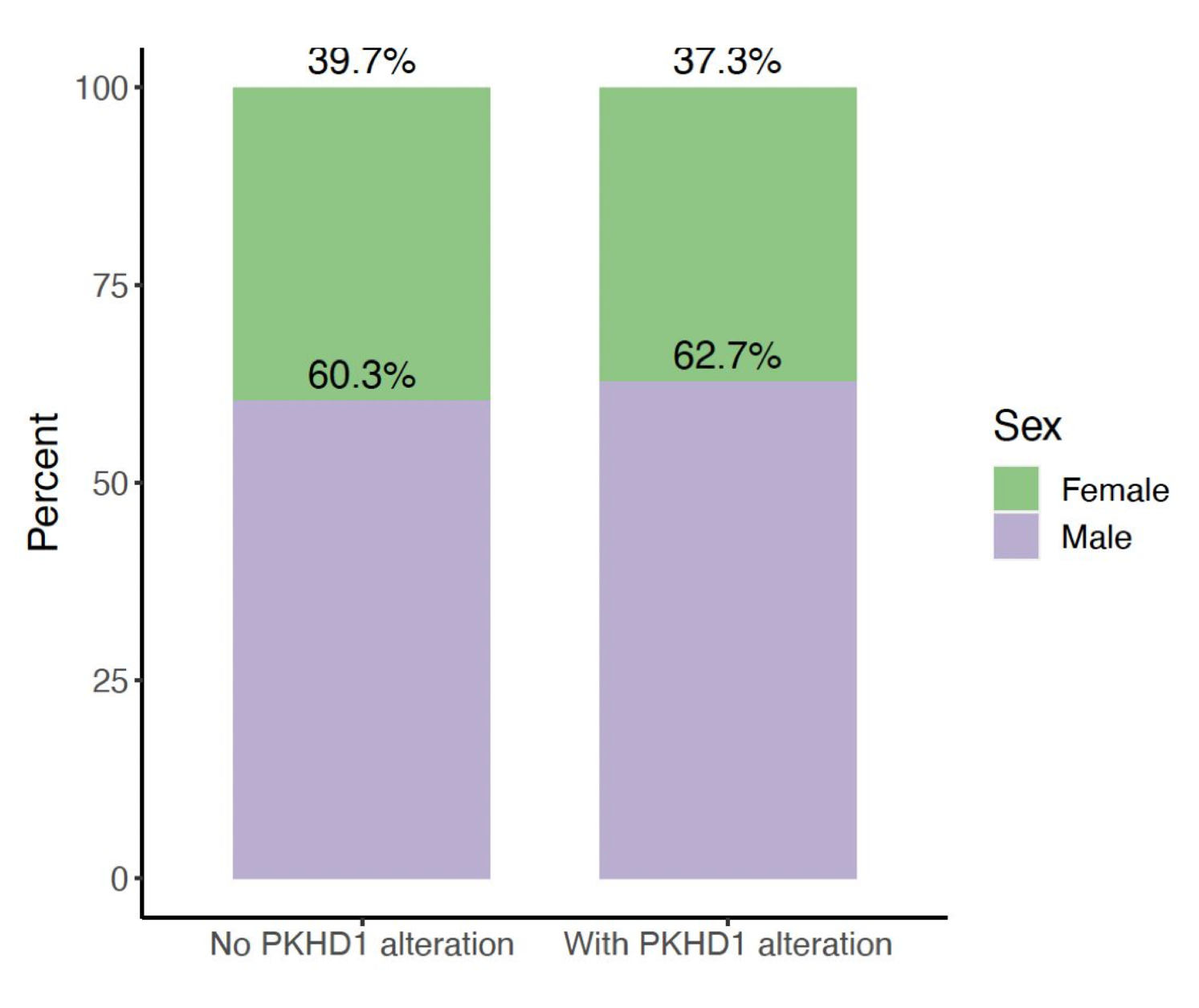


**Supplemental Figure 3. Sex comparison between PKHD1 and wild-type groups.**


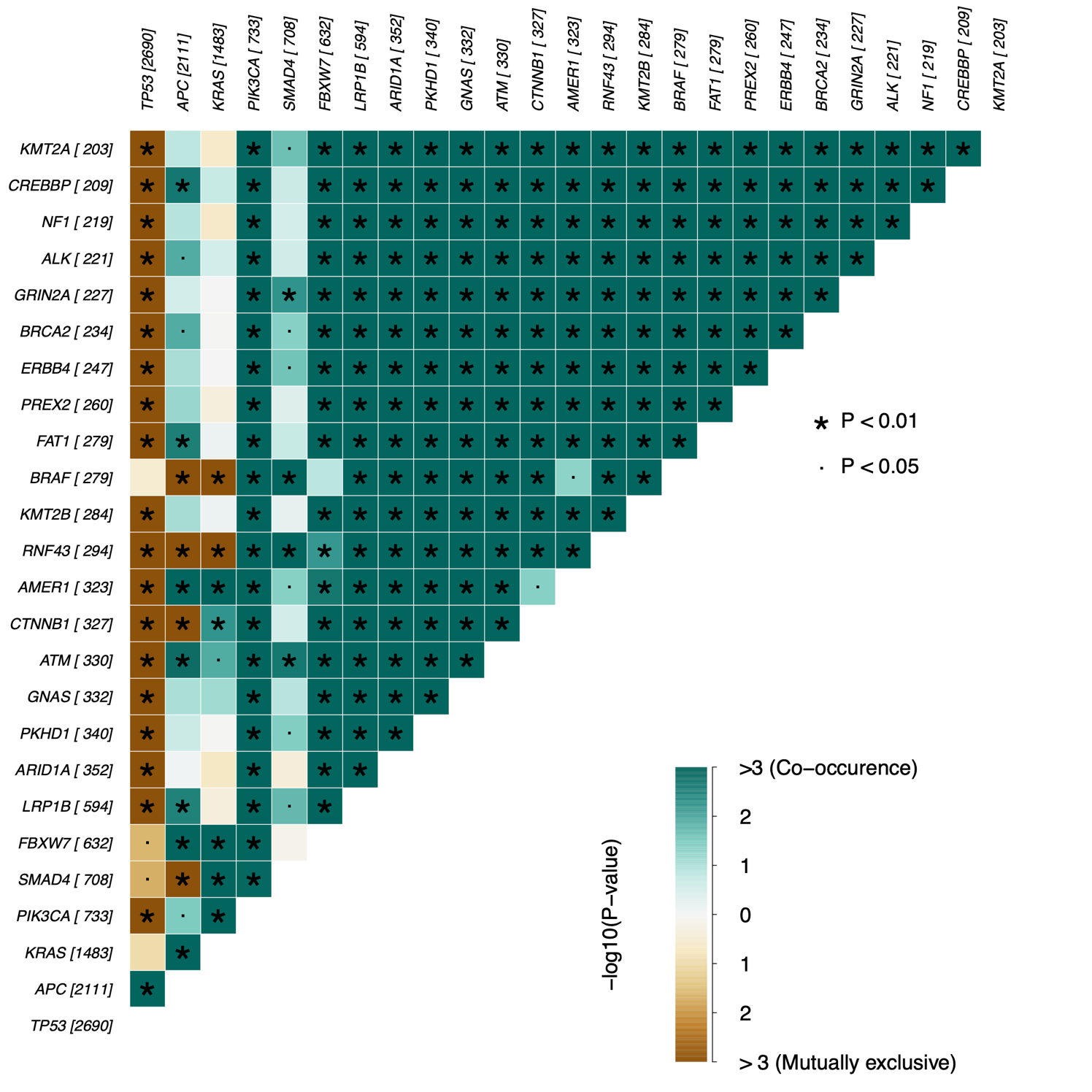


**Supplemental Figure 4.** Somatic co-alterations in colon cancer patients. The Co-occurrence of mutually exclusive mutations of the top 15 genes in colon cancer is shown.


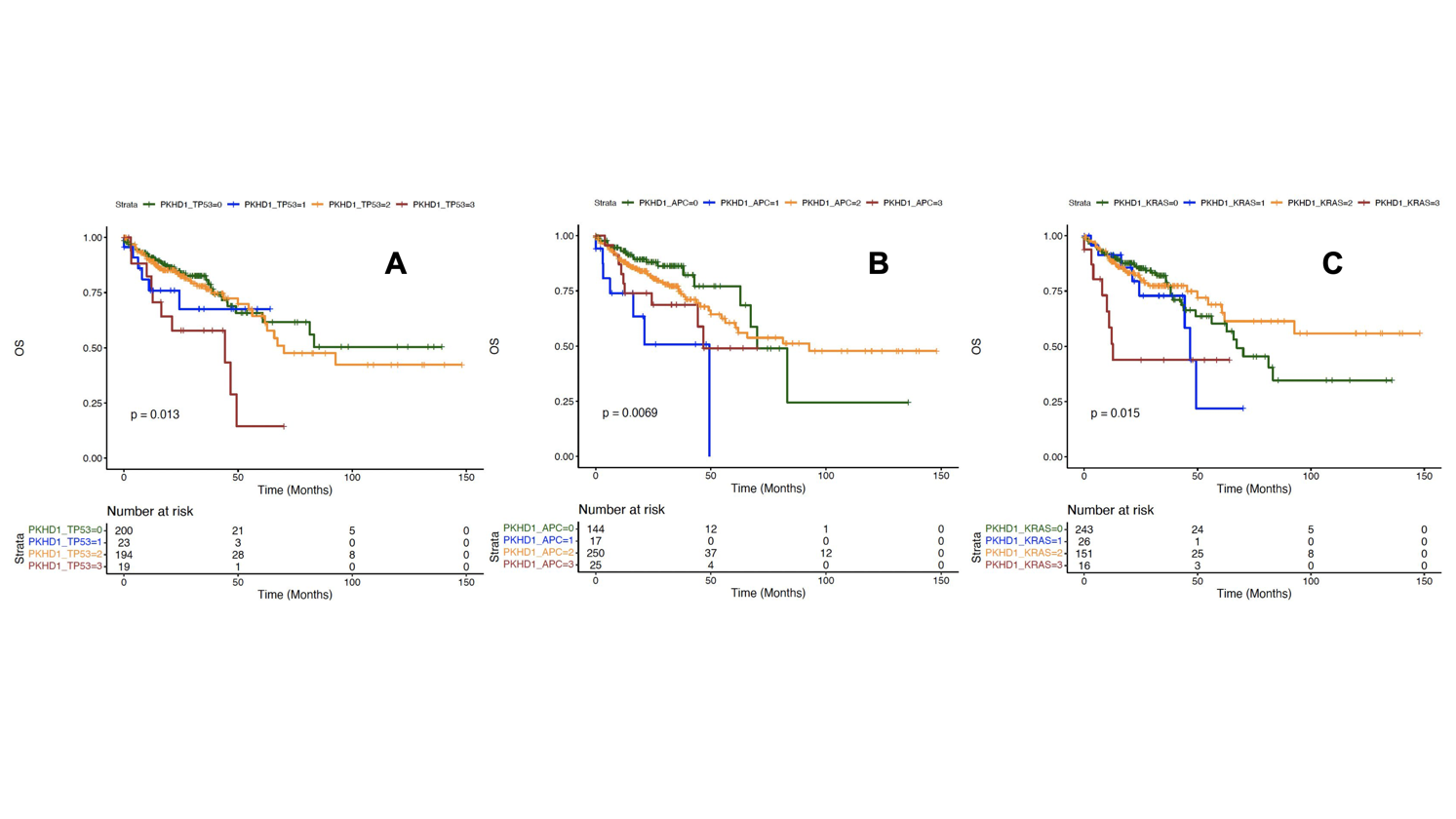


**Supplemental Figure 5. Additional overall survival analysis for PKHD1 patients.** Overall survival analysis of *PKHD1* co-occurred with A) *TP53*, B) *APC*, and C) *KRAS*. "*PKHD1*_*GENE*=0" represents samples wild type for both *PKHD1* and the respective gene; "*PKHD1*_*GENE*=1" for mutations in both *PKHD1* and the respective gene; "*PKHD1*_*GENE*=2" for *PKHD1* wild type and mutated respective gene; and "*PKHD1*_*GENE*=3" for *PKHD1* mutations with the respective gene wild type. Where “*GENE*” represents each of the genes (*TP53*, *APC*, *KRAS*) analyzed in conjunction with *PKHD1*.

## Supplementary Tables

**Table S1. Summary of characters of in-house colon cancer patients.**

| Total, n | | 3702 |
| --- | --- | --- |
| Age, Mean ± SD | | 58.5 ± 12.29 |
| TMB, Mean ± SD | | 16.67 ± 47.55 |
| Sex, n (%) | Female | 1518(41.00%) |
|  | Male | 2184(59.00%) |
| Stage, n (%) | 0-I | 528(14.26%) |
|  | II | 1237(33.41%) |
|  | III | 1052(28.42%) |
|  | IV | 638(17.23%) |
|  | Unknown | 236(6.37%) |

**Table S2. Univariate Cox analysis of in-house colon cancer patients for prediction of overall survival.**

| Variables | | HR | 95% CI | p-value |
| --- | --- | --- | --- | --- |
| SEX | Female | reference | | |
|  | Male | 1.164 | (0.7804-1.736) | 0.456 |
| MSI | 0 (<19) | reference | | |
|  | 1($\geq$19) | 1.0243 | (0.5814-1.805) | 0.934 |
| AGE |  | 1.02 | (1.007-1.041) | 0.00498 |
| Stage | I | reference | | |
|  | II | 2 | (0.7843-5.266) | 0.1444 |
|  | III | 3.3 | (1.2832-8.542) | 0.0133 |
|  | IV | 9.4 | (3.6578-23.967) | 3.10E-06 |
| TMB |  | 1.001444 | (0.9958-1.007) | 0.616 |
| *PKHD1* | 0 | reference | | |
|  | 1 | 2.0323 | (1.214-3.403) | 0.00702 |
| *KRAS* | 0 | reference | | |
|  | 1 | 0.996757 | (0.666-1.492) | 0.987 |
| *TP53* | 0 | reference | | |
|  | 1 | 1.2112 | (0.8124-1.806) | 0.347 |
| *APC* | 0 | reference | | |
|  | 1 | 1.1695 | (0.7517-1.82) | 0.488 |

**Table S3. Association between cancer stage and PKHD1 mutation status in in-house cohort.**

| *PKHD1* status | Stage 1 | Stage 2 | Stage 3 | Stage 4 | Unknown | Total | *p (chi-square)* |
| --- | --- | --- | --- | --- | --- | --- | --- |
| *PKHD1* mutate | 28(5.30%) | 253(20.45%) | 84(7.98%) | 48(7.52%) | 11(4.45%) | 424 | 2.20E-16 |
| *PKHD1* wild type | 500(94.70%) | 984(79.55%) | 968(92.02) | 590(92.48%) | 236(95.55%) | 3278 |  |
| Total | 528 | 1237 | 1052 | 638 | 247 | 3702 |  |

**Table S4. Association between cancer stage and *PKHD1* mutation status in TCGA COAD cohort.**

| *PKHD1* status | Stage 1 | Stage 2 | Stage 3 | Stage 4 | Unknown | Total | *p (chi-square)* |
| --- | --- | --- | --- | --- | --- | --- | --- |
| *PKHD1* mutate | 3(4.00%) | 32(18.93%) | 9(7.32%) | 4(6.56%) | 2(25%) | 50 | 2.51E-05 |
| *PKHD1* wild type | 72(96.00%) | 137(81.07%) | 114(92.68%) | 57(93.44%) | 6(75%) | 386 |  |
| Total | 75 | 169 | 123 | 61 | 8 | 436 |  |

# Table S5. P-value from comparing infiltrating of a complete list of 39 cell types between PKHD1 and wild-type groups.

| Type | P value |
| --- | --- |
| B cell plasma_XCELL | 0.000386253 |
| Macrophage_XCELL | 0.003667977 |
| T cell CD4+ naive_XCELL | 0.007557254 |
| B cell_XCELL | 0.00777695 |
| T cell CD8+ central memory_XCELL | 0.008275094 |
| immune score_XCELL | 0.009191778 |
| Common lymphoid progenitor_XCELL | 0.009993711 |
| Plasmacytoid dendritic cell_XCELL | 0.011644586 |
| Hematopoietic stem cell_XCELL | 0.018369589 |
| T cell CD4+ memory_XCELL | 0.019396576 |
| Macrophage M2_XCELL | 0.026220346 |
| Class-switched memory B cell_XCELL | 0.028951014 |
| T cell gamma delta_XCELL | 0.069927217 |
| T cell CD4+ Th1_XCELL | 0.087246874 |
| T cell CD4+ Th2_XCELL | 0.091644523 |
| T cell regulatory (Tregs)_XCELL | 0.096070799 |
| B cell memory_XCELL | 0.114404656 |
| Granulocyte-monocyte progenitor_XCELL | 0.140824336 |
| T cell CD8+_XCELL | 0.165082781 |
| T cell CD4+ (non-regulatory)_XCELL | 0.181826281 |
| Macrophage M1_XCELL | 0.197786148 |
| T cell CD4+ central memory_XCELL | 0.201410779 |
| Myeloid dendritic cell activated_XCELL | 0.205676462 |
| stroma score_XCELL | 0.273341511 |
| T cell NK_XCELL | 0.286174177 |
| Monocyte_XCELL | 0.340231846 |
| Endothelial cell_XCELL | 0.361711182 |
| microenvironment score_XCELL | 0.391149506 |
| Mast cell_XCELL | 0.444968055 |
| Cancer associated fibroblast_XCELL | 0.450908279 |
| NK cell_XCELL | 0.469172357 |
| T cell CD4+ effector memory_XCELL | 0.48293554 |
| T cell CD8+ effector memory_XCELL | 0.5689495 |
| Common myeloid progenitor_XCELL | 0.63418737 |
| Myeloid dendritic cell_XCELL | 0.718247045 |
| T cell CD8+ naive_XCELL | 0.826547842 |
| Neutrophil_XCELL | 0.958383534 |
| Eosinophil_XCELL | 0.970303396 |
| B cell naive_XCELL | 0.976266696 |
